# Mapping the burden of cholera in sub-Saharan Africa and implications for control: an analysis of data across geographical scales

**DOI:** 10.1016/S0140-6736(17)33050-7

**Published:** 2018-05-12

**Authors:** Justin Lessler, Sean M Moore, Francisco J Luquero, Heather S McKay, Rebecca Grais, Myriam Henkens, Martin Mengel, Jessica Dunoyer, Maurice M'bangombe, Elizabeth C Lee, Mamoudou Harouna Djingarey, Bertrand Sudre, Didier Bompangue, Robert S M Fraser, Abdinasir Abubakar, William Perea, Dominique Legros, Andrew S Azman

**Affiliations:** a Department of Epidemiology, Johns Hopkins Bloomberg School of Public Health, Baltimore, MD, USA; b Department of International Health, Johns Hopkins Bloomberg School of Public Health, Baltimore, MD, USA; c Department of Biological Sciences, University of Notre Dame, Notre Dame, IN, USA; d Eck Institute for Global Health, University of Notre Dame, Notre Dame, IN, USA; e Epicentre, Paris, France; f Médecins Sans Frontières International Office, Brussels, Belgium; g Agence de Médecine Préventive, Paris, France; h UNICEF West and Central Africa Regional Office, Dakar, Senegal; i Epidemiology Unit, Malawi Ministry of Health, Lilongwe, Malawi; j Global Infectious Diseases, Georgetown University, Washington, DC, USA; k WHO Office for Africa, Brazzaville, Republic of the Congo; l European Centre for Disease Prevention and Control, Stockholm, Sweden; m Ministry of Health, Kinshasa, Democratic Republic of the Congo; n University of Kinshasa, Kinshasa, Democratic Republic of the Congo; o International Federation for the Red Cross and Red Crescent Societies, Geneva, Switzerland; p WHO Office for the Eastern Mediterranean, Cairo, Egypt; q WHO, Geneva, Switzerland; r Médecins sans Frontières, Geneva, Switzerland

## Abstract

**Background:**

Cholera remains a persistent health problem in sub-Saharan Africa and worldwide. Cholera can be controlled through appropriate water and sanitation, or by oral cholera vaccination, which provides transient (∼3 years) protection, although vaccine supplies remain scarce. We aimed to map cholera burden in sub-Saharan Africa and assess how geographical targeting could lead to more efficient interventions.

**Methods:**

We combined information on cholera incidence in sub-Saharan Africa (excluding Djibouti and Eritrea) from 2010 to 2016 from datasets from WHO, Médecins Sans Frontières, ProMED, ReliefWeb, ministries of health, and the scientific literature. We divided the study region into 20 km × 20 km grid cells and modelled annual cholera incidence in each grid cell assuming a Poisson process adjusted for covariates and spatially correlated random effects. We combined these findings with data on population distribution to estimate the number of people living in areas of high cholera incidence (>1 case per 1000 people per year). We further estimated the reduction in cholera incidence that could be achieved by targeting cholera prevention and control interventions at areas of high cholera incidence.

**Findings:**

We included 279 datasets covering 2283 locations in our analyses. In sub-Saharan Africa (excluding Djibouti and Eritrea), a mean of 141 918 cholera cases (95% credible interval [CrI] 141 538–146 505) were reported per year. 4·0% (95% CrI 1·7–16·8) of districts, home to 87·2 million people (95% CrI 60·3 million to 118·9 million), have high cholera incidence. By focusing on the highest incidence districts first, effective targeted interventions could eliminate 50% of the region's cholera by covering 35·3 million people (95% CrI 26·3 million to 62·0 million), which is less than 4% of the total population.

**Interpretation:**

Although cholera occurs throughout sub-Saharan Africa, its highest incidence is concentrated in a small proportion of the continent. Prioritising high-risk areas could substantially increase the efficiency of cholera control programmes.

**Funding:**

The Bill & Melinda Gates Foundation.

## Introduction

Cholera is one of the oldest known infectious diseases. Seven times in recorded history, cholera has emerged from its presumed natural home near the Bay of Bengal and spread globally. Although each of the previous six pandemics receded, the present (seventh) pandemic has persisted for over 50 years.^1^ Sub-Saharan Africa is one region in which cholera has persisted, with both endemic regions (ie, regions with persistent incidence) and sporadic, high-mortality epidemics, which can amplify already complex crises such as famines and civil conflict.^2^ Between 2000 and 2015, 52 812 (83%) of 63 658 cholera deaths reported by WHO occurred in sub-Saharan Africa,^3^ although this percentage is probably an overestimate because much of south Asia's cholera burden goes unreported.^4^

Spurred by large outbreaks in Yemen, Tanzania, Haiti, and elsewhere in the past decade and the availability of new methods to control cholera outbreaks, the WHO-led Global Task Force on Cholera Control has laid out a roadmap for ending cholera as a public health threat by 2030.^5^ Access to safe water, appropriate sanitation, and hygiene (WaSH) remains the foundation of sustained cholera control, but a new generation of easy-to-use oral cholera vaccines (OCVs) also have an important role. These vaccines are socially acceptable, safe, and effective for prevention of cholera for at least 3 years after administration.^6^ OCVs can curb transmission in the short term, preventing death and disease while crucial improvements to infrastructure are made. However, although supplies are increasing every year, the number of OCV doses available (∼17 million produced in 2017)^7^ remains too low for large, generalised campaigns. Even if adequate supplies become available, broad use of a vaccine with only transient protection is unlikely to be cost-effective in populations in which few are at high risk, and high population turnover in vulnerable populations might limit its long-term effect. Thus, integrated strategies for efficient use of OCVs and WaSH are needed.

**Research in context**

**Evidence before this study**

We searched PubMed for relevant studies published between Sept 25, 2012, and Sept 25, 2017, using the terms “cholera burden Africa”. Of 27 results, 24 were either not about cholera burden or were about cholera in a particular country or context. The remaining three were a study by Ali and colleagues, who estimated global burden of cholera in endemic countries; a study by Moore and colleagues, who explored the effect of El Niño on the distribution of cholera in Africa; and a review of cumulative cases of cholera in Africa reported to WHO at the country level between 1970 and 2011 (3·2 million cases), which also includes incidence estimates based on data from the study by Ali and colleagues. Ali and colleagues applied cholera incidence rates from three sentinel sites in India, Indonesia, and Mozambique to at-risk populations (classed as those with no access to improved sanitation) matched by WHO mortality strata to provide country-level estimates of cholera incidence in endemic countries. They estimated that 1·3 billion people (550 million in sub-Saharan Africa) are at risk for cholera and 2·9 million cases (1·7 million in sub-Saharan Africa) occur per year.

**Added value of this study**

Previous estimates of populations at risk, based on national-level data, obscure heterogeneities in cholera epidemiology at the subnational level that are likely to have important implications for cholera control. In our study we used, to our knowledge, the largest combined dataset on sub-Saharan African cholera epidemiology to substantially increase our understanding of the distribution of the disease in the region. We found that 21·7 million people (95% CrI 19·8 million to 23·7 million) in sub-Saharan Africa live in areas of high-cholera incidence (20 km × 20 km grid cells in which more than one in 1000 people are reported as infected with cholera each year), and 87·2 million (95% CrI 60·3 million to 118·9 million) live in districts with high incidence. An additional 252·4 million (169·0 million to 351·9 million) people live in districts with moderate incidence (≤1 case per 1000 people and >1 case per 10 000 people per year) and 177·6 million (112·8 million to 248·7 million) live in districts with mild incidence (≤1 case per 10 000 people and >1 case per 100 000 people per year). Our results show that cholera incidence is concentrated in high-burden hotspots, and we project that by targeting districts in order of their cholera incidence (from highest to lowest), an effective cholera intervention (eg, vaccination campaigns combined with improved water and sanitation) could eliminate 50% of reported cholera in the region by covering less than 4% of the population (∼35 million people). Even finer scale targeting of interventions could further improve the efficiency of use of cholera interventions. 55·9 million (64%) of 86·9 million people living in high-incidence districts live in areas where cholera incidence follows an endemic pattern.

**Implications of all available evidence**

Cholera remains a substantial threat to human health in sub-Saharan Africa and throughout the world, particularly in the poorest and most vulnerable communities. In October, 2017, the WHO-led Global Task Force on Cholera Control released a roadmap for ending cholera as a public health threat by 2030. To accomplish this goal, the tools at our disposal need to be used efficiently: improved water, sanitation, and hygiene and oral cholera vaccine, particularly given resource constraints and insufficient supplies of vaccine (∼17 million doses in 2017). Detailed mapping of cholera incidence can help us to best use the tools at our disposal and is an essential component in tracking our progress in the fight against cholera. However, substantial challenges to the characterisation of global burden remain, including expansion of these analyses to south Asia and other high-incidence regions and improvement of cholera surveillance globally.

Therefore, to reach the goal of ending cholera as a public health threat by 2030, development and implementation of methods to identify and appropriately target high-risk populations in sub-Saharan Africa and other high-burden regions (eg, south Asia) are essential. With this goal in mind, we used data on cholera incidence from 2010 to 2016 over several spatial scales and data on water and sanitation coverage to create high-resolution maps of cholera burden throughout sub-Saharan Africa.^8^, ^9^ Using these maps, we estimated the number of people living in areas where there is a high risk of acquiring symptomatic cholera and examined how geographical targeting could lead to more efficient interventions. We further characterised variations in cholera epidemiology throughout the region that are likely to have an effect on cholera control.

## Methods

### Data sources

We obtained cholera incidence data from sub-Saharan Africa from 2010 to 2016 from WHO, Médecins Sans Frontières, ProMED, ReliefWeb, ministries of health, and published versions of national surveillance data when available (already known to the authors and through literature searches; appendix). Data were input into a standard schema that enabled flexible entry of data spanning several reporting periods and case definitions (appendix). To estimate annual incidence rates, sub-annual data for each unique location were aggregated to an annual total. Most cholera cases are not confirmed clinically; hence, analyses are based on reports of suspected cholera. Summaries of datasets and instructions for requesting access are available from the Johns Hopkins Bloomberg School of Public Health's Cholera dynamics website.

### Mapping methods

Using a previously published Bayesian modelling framework,^9^ we produced maps of cholera incidence rates from 2010 to 2016 by integrating reported cholera case data from several spatial and temporal scales with key environmental and socioeconomic risk factors (appendix). We divided the study region (sub-Saharan Africa, excluding Djibouti and Eritrea) into 20 km × 20 km grid cells and modelled annual cholera incidence in each grid cell using a log-linear regression with covariates and spatially correlated random effects. Each observation was mapped to the underlying grid cells (using the raster package^10^ in R) and assumed to be an independent realisation from a Poisson distribution such that the expected number of cases in an observation area was the sum of the expected number of cases for all grid cells in the observation area. This method means that duplicate observations of the same grid cell at the same time (eg, a cell included in both a district and province for which cholera incidence was reported) are classed as separate imperfect observations of the true number of cases in that grid cell, rather than non-overlapping case reports (ie, cases reported in both datasets are not double counted). For point-based observations, such as geo-located case data or cases from a single neighbourhood or refugee camp, we used the grid cell containing that global positioning system point. Grid cells were included in the administrative regions where their centroid (ie, centre point) fell. We defined province as the first subdivision of a country and district as the second subdivision of a country after province, state, or region in GADM 2.8.

We included ecological covariates known to affect cholera risk: proportion of the population with access to improved drinking water (modified WHO/UNICEF Joint Monitoring Program definition),^8^ proportion of the population with access to improved sanitation (modified WHO/UNICEF Joint Monitoring Program definition),^8^ population density,^11^ distance to nearest coastline, and distance to nearest major waterbody (ie, lake, river, or reservoir). We did not obtain water and sanitation data for Djibouti and Eritrea, and these countries were excluded from all analyses (appendix). All covariates were resampled to 20 km × 20 km grid cells.

We used Markov chain Monte Carlo methods with Rstan 2.7 for inference. We ran 2000 iterations of the sampler with four parallel chains and assessed convergence with the Gelman-Rubin convergence statistic .^12^

### Estimation of high-risk populations and cases averted by simple interventions

We used maps of estimated annual cholera incidence to identify grid cells in which annual cholera incidence was mild (≤1 case per 10 000 people and >1 case per 100 000 people), moderate (≤1 case per 1000 people and >1 case per 10 000 people), or high (>1 case per 1000 people; appendix); we did not formally classify incidence of less than or equal to one case per 100 000 people. The number of people living in these grid cells was then aggregated by district and country in each Markov chain Monte Carlo iteration. Districts were categorised as having the highest grid cell incidence level (high, moderate, or mild) experienced by at least 100 000 residents or 10% of the population of that district. We calculated summary statistics (eg, medians and means) and 95% credible intervals (CrIs) by sampling from the posterior distribution of incidence rates.

We estimated the extent of urban areas using satellite-derived land cover estimates from MODIS.^13^ We categorised grid cells as urban if more than 1% of the cell's land cover in 2013 was classified as a developed or built environment, and as rural otherwise.

When estimating the effect of targeted interventions (ie, OCVs or WaSH interventions aimed at the population of a specific geographical area), we assumed that mean incidence from 2010 to 2016 was reflective of expected incidence over the hypothetical intervention period. Unless otherwise noted, we assumed that an integrated set of interventions would be effective at reducing cholera to negligible levels in those targeted. When estimating the potential reductions in incidence from OCV deployments, we assumed that only people aged 1 year and older were eligible (calculated from regional birth and death rates^14^) and that 72% of these were protected for 3 years after vaccination, on the basis of an estimated vaccine effectiveness of 83% in the first year and 69% in the third year.^5^ We assumed no vaccine protection after the third year.^6^

To characterise differences in cholera epidemiology beyond incidence, we plotted overall incidence versus year-to-year variation in incidence at the national level for all of sub-Saharan Africa, and at the district level for two exemplar countries (Nigeria and the Democratic Republic of Congo). Calculation of variance requires a longer time series than that used in our main analysis, hence this analysis was based on national-level incidence estimates reported for 2000–15,^4^ and sub-national time series for Nigeria (2004–14) and the Democratic Republic of Congo (2000–16; these data are included in our main analysis when appropriate). Year-to-year variation is characterised by the coefficient of variation (CV; SD of annual incidence divided by the mean annual incidence).

### Data sharing

Metadata for all data used in these analyses are available online. A portion of the data are available directly for download, whereas others were shared under data-sharing agreements that prohibit distribution without prior consent of the data owner. We will facilitate access to any non-publicly available data; please contact cholera_data@jhu.edu. Source code for the model is available online.

### Role of the funding source

The funder of the study had no role in study design, data collection, data analysis, data interpretation, or writing of the report. JL and ASA had full access to all the data in the study and had final responsibility for the decision to submit for publication.

## Results

**Figure 1:**
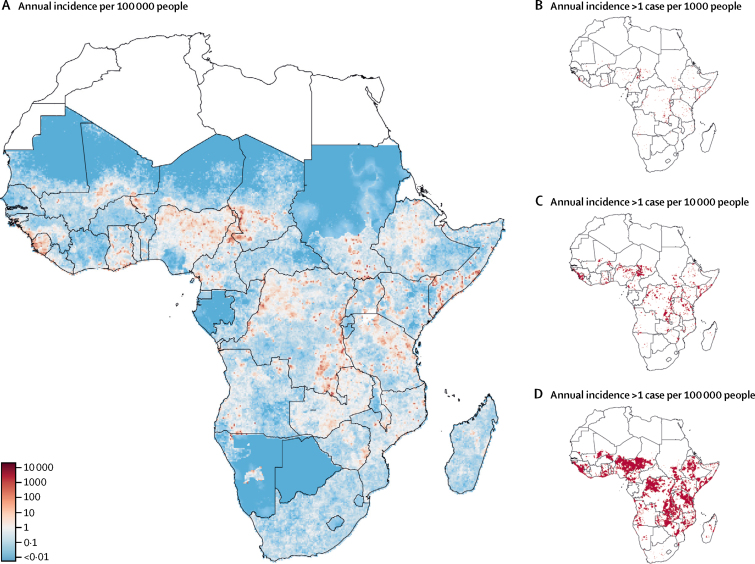
Mean annual cholera incidence (A) Mean annual cholera incidence per 100 000 people in sub-Saharan Africa between 2010 and 2016, and (B) locations with mean annual incidence of more than one per 1000 people, (C) more than one per 10 000 people, or (D) more than one per 100 000 people.

In our analyses, we included 279 datasets representing epidemiological time series of 1–7 years in length. These datasets include data for 2283 different, though sometimes nested, locations in 37 countries in sub-Saharan Africa (appendix). Our study area (ie, excluding Djibouti and Eritrea) had a population of 988, 919 755 people. Within our study area, 27·5% (272 423 332 of 988 919 755) of the population were classed as urban—marginally lower than the 2016 UN and World Bank estimate of 38%.^14^, ^15^ Across sub-Saharan Africa (excluding Djibouti and Eritrea), a mean of 141 918 cholera cases (95% CrI 141 538–146 505) were reported annually, although year-to-year variation was substantial, as indicated by the calculated CVs (data not shown). We estimated that 21 695 308 people (19 757 461–23 654 156) in sub-Saharan Africa live in 20 km × 20 km grid cells in which average annual cholera incidence is greater than one in 1000 people (high incidence; figure 1). 61·1% (54·2–66·4) of this population live in rural areas, which is similar to that in the sub-Saharan African population overall (62%).^15^ 65 471 944 people (55 335 356–70 896 060) live in grid cells with moderate incidence, and 126 253 539 people (116 107 402–139 827 994) live in grid cells with mild incidence. 543 554 976 people (482 466 484–588 554 996) live in areas with negligible incidence of reported cholera (<1 per 1 million people per year), constituting 55·0% of the study area population.

**Figure 2:**
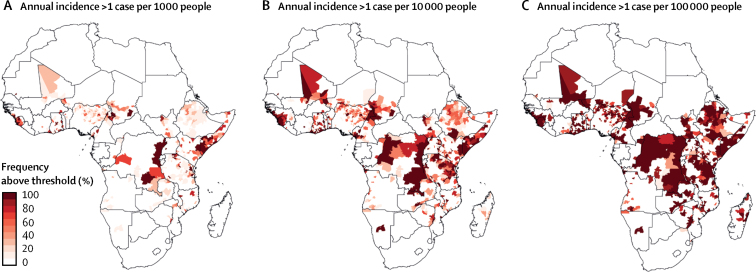
Districts with mean annual cholera incidence above certain thresholds (A) Districts with mean annual cholera incidence of more than one case per 1000 people, (B) more than one case per 10 000 people, and (C) more than one case per 100 000 people. Districts with a mean of fewer than five cases annually are excluded. The colour scale represents the percentage of model iterations (ie, posterior draws) for which incidence exceeds the threshold, with darker shaded districts being over the threshold in a higher percentage of Markov chain Monte Carlo iterations.

We estimated that 151 of 3751 districts (4·0%, 95% CrI 1·7–16·8) in our study area, home to 87·2 million people (95% CrI 60·3 million to 118·9 million), can be classified as having high incidence of cholera (figure 2). 168·3 million (116·4 million to 229·4 million) OCV doses would be needed to vaccinate all eligible individuals living in these high-risk districts, which is substantially more vaccine than has been produced since the establishment of the global stockpile in 2013.^7^ We estimated that vaccinating all eligible individuals living in high-risk districts would directly prevent a mean of 156 536 cases (121 094–194 640) over a 3-year period. Extending vaccination to eligible individuals living in both moderate-risk and high-risk districts could directly prevent an estimated 239 518 cases (173 581–379 223), but 511·6 million (396·4 million to 635·8 million) doses of vaccine would be needed, which is more than 30 times the estimated global production in 2017.^7^ Indirect effects of vaccination campaigns (ie, herd protection) could protect more individuals, even with imperfect coverage, although there is conflicting evidence as to the nature and spatial scale of indirect effects.^16^, ^17^

**Figure 3:**
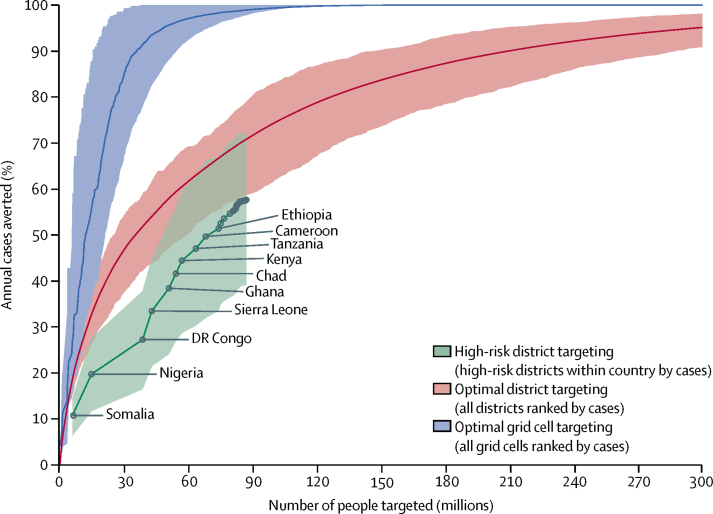
Annual cholera cases in sub-Saharan Africa averted as a function of the number of people targeted with an ideal intervention or mix of interventions The optimum grid cell targeting curve (blue) represents a strategy targeting all 20 km × 20 km grid cells in rank order by number of cases. The optimum district targeting curve (red) represents a strategy targeting all districts in rank order by number of cases regardless of country. The green curve represents a more realistic and practical strategy that targets all high-risk districts in each country at once, with countries ranked by the number of cases prevented. Lines are the mean values and shading shows the 95% credible intervals. Strategies targeting grid cells or districts by ranked incidence instead of number of cases are presented in the appendix.

Substantial strides could be made towards cholera elimination with a fraction of the resources by prioritising those areas at highest risk. For example, consider the number of people who would need to be effectively targeted by an integrated cholera control programme to prevent 50% of the cholera cases in sub-Saharan Africa. If we focus interventions at the resolution of our maps (20 km × 20 km grid cells), targeting the areas in order of their number of cases from 2010 to 2016 from highest to lowest, incidence could be reduced by 50% by targeting areas containing 11·9 million people (95% CrI 5·6 million to 21·1 million), which is 1·2% of the study population (figure 3). If the same strategy was used to target districts, areas containing 35·3 million people (26·3 million to 62·0 million; figure 3), which consitutue 3·6% (95% CrI 2·7–6·3) of our study population, would be targeted. An alternative strategy ranking by incidence rather than the number of cases led to similar reductions (appendix). A similar approach could be used to make optimum use of existing resources. For example, if 20 million OCV doses were used in the highest incidence districts, 121 637 (29%) of 425 754 cholera cases (83 620–182 694) could be prevented over 3 years from direct effects of the vaccine.

In practice, targeting the high-incidence areas irrespective of geography, population size, accessibility, or other factors is probably not feasible because of logistical challenges. A more practical strategy might be to prioritise countries on the basis of the number of cases that could be averted if all high-incidence districts in the country were targeted, then launch country-specific programmes aimed at those districts. Using this approach, 38·4% of cholera cases could be prevented by covering 50·8 million people (95% CrI 39·7 million to 62·8 million) in five countries: Somalia, Nigeria, Democratic Republic of the Congo, Sierra Leone, and Ghana (figure 3).

**Figure 4:**
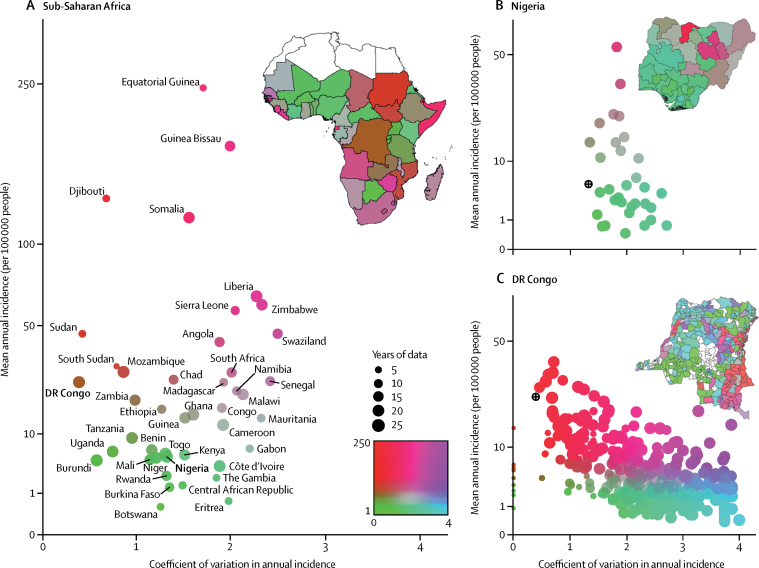
Cholera incidence versus the coefficient of variation of the annual incidence Mean annual reported cholera incidence per 100 000 people versus the coefficient of variation of the annual incidence from 2000 to 2015 for 50 African countries based on reports to WHO (A),^3^ and from 2004 to 2014 for districts in Nigeria (B; states) and from 2000 to 2016 for the Democratic Republic of the Congo (C; zone de santé) using annual aggregated data for each country. Colouring of points and map areas corresponds to the position on the scatter plot, as shown in the bottom right inset in (A), to allow easier mapping between country maps and *x*–*y* plots. The size of the points correspond to the number of years that the country reported data to WHO. Countries and districts with a mean annual incidence of zero are dark green in the sub-maps, but are not plotted in the scatter plots. Black circles and crosses in (B) and (C) represent each country's position in (A). National-level data were available for both Djibouti and Eritrea; white areas on maps correspond to areas where data were not available.

To better characterise the prevailing cholera dynamics on this endemic–epidemic spectrum (in which endemic dynamics are associated with a low CV and epidemic dynamics are associated with a high CV), we plotted the CV in year-to-year cholera incidence for each country against their mean yearly incidence per 100 000 population over that same period (figure 4). This revealed a spectrum of cholera dynamics that included both high-incidence countries with endemic dynamics (eg, Democratic Republic of the Congo) and those with epidemic dynamics (eg, Guinea-Bissau). As with incidence, consideration of subnational epidemiological patterns that might have important policy implications is crucial. For instance, although, as a whole, Nigeria had consistently low cholera incidence, the eastern part of the country had large populations with high rates of epidemic cholera (figure 4). Likewise, the high incidence of endemic cholera in the Democratic Republic of the Congo was driven by districts in the east of the country, whereas other areas had a more epidemic pattern (figure 4). Unfortunately, for much of Africa, we do not have sufficient temporal data of high spatial resolution to classify all districts along this spectrum. However, assuming high-incidence districts within each country follow national dynamics, we estimate that 55·9 million people (95% CrI 38·8 million to 78·9 million) in Africa live in high-incidence districts with endemic (ie, predictable) cholera (CV <1·5), whereas 31·0 million (95% CrI 20·6 to 39·8 million) live in districts with more epidemic dynamics (CV >1·5).

## Discussion

By characterising the geographical distribution of cholera risk in sub-Saharan Africa, we show that cholera is pervasive throughout sub-Saharan Africa, and that more than 200 million people are living in areas (ie, 20 km × 20 km grid cells) with at least some cholera incidence. We found substantial heterogeneities within and between countries; most cholera incidence is concentrated disproportionately in a small proportion (<5%) of districts. This finding highlights the importance of hotspots of high cholera burden in driving cholera incidence and a crucial role for spatial targeting in cholera control. As global cholera control efforts are intensified, with the goal of eliminating cholera as a public health threat, this study provides important estimates of baseline cholera burden and methods for tracking our progress.

The utility of cholera mapping depends on alignment of results to the geographical scales at which public health policy is made and implemented. The precise scale of interest will depend on political boundaries and the intervention being considered. Because of the scope of our analysis, we focused on broad-scale interventions (eg, district level) that might be most relevant to population-level programmes such as OCV campaigns. By contrast, WaSH interventions will often be targeted according to the scale of water and sanitation infrastructure (eg, at towns or villages), and thus finer scale maps supplemented by detailed analysis of the local situation might be more informative.

Our analysis shows how the temporal distribution of cholera incidence varies substantially from country to country, and often also from district to district. Some areas have truly endemic cholera with high numbers of cases every year (eg, eastern Democratic Republic of the Congo). At the other extreme are areas where cholera incidence is concentrated in large outbreaks separated by many years of low activity. Analysis of variation is likely to help to ensure resources are used effectively. The distribution of cholera will change, but historic trends are usually the best evidence available about future disease incidence, and our confidence in future projections will be highest when past variation is low. Likewise, the best strategy for using OCV and WaSH interventions will vary depending on an area's position on the endemic–epidemic spectrum.

Mapping cholera incidence on the basis of reported cases has many limitations. Cholera is probably under-reported, particularly in rural areas. Likewise, many countries report incidence of acute watery diarrhoea as a proxy for cholera, but even in hyper-endemic areas as few as 20% of acute watery diarrhoea cases test positive for *Vibrio cholerae*.^18^ Although use of several independent data sources helped to reduce the risk of bias stemming from these issues in reporting of data, correcting for these biases remains an area of ongoing research. Improvements to cholera surveillance would also help, and are a key element of the global cholera control roadmap. Year-to-year variation in incidence does not give a complete picture of local cholera dynamics, and there are many patterns of incidence that are compatible with each position on figure 4 (eg, 3 years of high incidence followed by 12 years of low incidence could have the same variation as peaks in incidence every 5 years); only detailed analysis of local data can give a full picture of local dynamics. Furthermore, although we identified many regions of high cholera incidence where known risk factors such as infrastructure problems or displaced populations are present, this study was not designed to assess the extent to which local cholera incidence can be attributed to these factors. Another fundamental challenge is local variations in cholera epidemiology. In some areas, cholera incidence is likely to increase during rainy periods as a result of contamination of water supplies. In others, incidence is likely to increase during periods of drought, when people are forced to use unsafe water sources. Most challenging are outbreaks associated with disruptions to the local infrastructure; for instance, the increased incidence of cholera after interruption of the chlorinated water supply in Uvira, Democratic Republic of the Congo.^19^ Our inability to predict such infrastructure problems, combined with local differences in cholera epidemiology, is one reason why mapping methodologies based mainly on using models fit to relationships with environmental covariates have been less successful for cholera than for other diseases (eg, malaria and dengue). These infrastructure changes and local variations in cholera epidemiology might lead to substantial deviations from our maps in the distributions of cholera risk over time, both in terms of increased and decreased incidence. Hence, these maps should be updated over time, and are not a substitute for local investigations of cholera epidemiology when planning local control strategies.

The 87 million people living in districts in sub-Saharan Africa with high incidence of cholera will be best helped by improvements to local water and sanitation infrastructure that would provide broad benefits beyond this one disease and ensure sustained cholera elimination. However, experience tells us that these improvements will need substantial investment and can take years to be fully realised, and that success will be dependent on unpredictable political or economic conditions. Until that time, OCVs remain an important tool to prevent and control the spread of cholera. Although OCV supplies have increased substantially since establishment of the global stockpile^7^ and are likely to continue to increase, they will probably remain insufficient for broad untargeted use of the vaccine. The water and sanitation improvements that are the ultimate solution to global cholera are likely to be most effective if they are geographically targeted. Regardless of how reductions in incidence are achieved, targeting people at high risk can have effects across much larger populations. As with sub-Saharan Africa, targeted approaches are likely to be an essential component of cholera control programmes in other high-burden regions, such as south Asia, Haiti, and the Middle East. Hence, fine-scale incidence mapping exercises such as this have an important role to play globally in maximising the benefits of scant resources, forecasting demand for vaccines and other supplies, and tracking progress in the fight against cholera.

For the **Cholera dynamics website** see http://www.iddynamics.jhsph.edu/projects/cholera-dynamics

## References

[1] Echenberg M (2011). Africa in the time of cholera: a history of pandemics from 1817 to the present.

[2] Bhattacharya S, Black R, Bourgeois L (2009). Public health. The cholera crisis in Africa. Science.

[3] WHO Global Health Observatory data repository. By category. Number of reported deaths, data by country. http://apps.who.int/gho/data/node.main.176?lang=en.

[4] WHO Global health observatory (GHO) data. Number of reported cholera cases. http://www.who.int/gho/epidemic_diseases/cholera/cases_text/en/.

[5] Global Task Force on Cholera Control Ending cholera: a global roadmap to 2030. http://www.who.int/cholera/publications/global-roadmap/en/.

[6] Bi Q, Ferreras E, Pezzoli L, for the Oral Cholera Vaccine Working Group of The Global Task Force on Cholera Control (2017). Protection against cholera from killed whole-cell oral cholera vaccines: a systematic review and meta-analysis. Lancet Infect Dis.

[7] Pezzoli L, on behalf of the Oral Cholera Vaccine Working Group of the Global Task Force for Cholera Control (2017). Deployments from the oral cholera vaccine stockpile, 2013–2017. Wkly Epidemiol Rec.

[8] Pullan RL, Freeman MC, Gething PW, Brooker SJ (2014). Geographical inequalities in use of improved drinking water supply and sanitation across sub-Saharan Africa: mapping and spatial analysis of cross-sectional survey data. PLoS Med.

[9] Moore SM, Azman AS, Zaitchik BF (2017). El Niño and the shifting geography of cholera in Africa. Proc Natl Acad Sci USA.

[10] Hijmans RJ (2015). raster: geographic data analysis and modeling. http://CRAN.R-project.org/package=raster.

[11] Tatem AJ (2017). WorldPop, open data for spatial demography. Sci Data.

[12] Gelman A, Rubin DB (1992). Inference from iterative simulation using multiple sequences. Stat Sci.

[13] Friedl MA, Sulla-Menashe D, Tan B (2010). MODIS Collection 5 global land cover: algorithm refinements and characterization of new datasets. Remote Sens Environ.

[14] Bank W (2015). World development indicators 2015.

[15] UN (2014). World urbanization prospects 2014: highlights.

[16] Ali M, Debes AK, Luquero FJ (2016). Potential for controlling cholera using a ring vaccination strategy: re-analysis of data from a cluster-randomized clinical trial. PLoS Med.

[17] Ali M, Sur D, You YA (2013). Herd protection by a bivalent killed whole-cell oral cholera vaccine in the slums of Kolkata, India. Clin Infect Dis.

[18] Alam M, Hasan NA, Sadique A (2006). Seasonal cholera caused by *Vibrio cholerae* serogroups O1 and O139 in the coastal aquatic environment of Bangladesh. Appl Environ Microbiol.

[19] Jeandron A, Saidi JM, Kapama A (2015). Water supply interruptions and suspected cholera incidence: a time-series regression in the Democratic Republic of the Congo. PLoS Med.

