# Supplementary material for: Mapping the burden of cholera in sub-Saharan Africa and implications for control: an analysis of data across geographical scales
Source: Lancet. 2018 May 12;391(10133):1908–15. doi: 10.1016/S0140-6736(17)33050-7 (PMC5946088; doi:10.1016/S0140-6736(17)33050-7)
Supplement: Supplementary appendix [file mmc1.pdf]

THE LANCET

Supplementary appendix

This appendix formed part of the original submission and has been peer reviewed. We post it as supplied by the authors.

Supplement to: Lessler J, Moore SM, Luquero FJ, et al. Mapping the burden of cholera in sub-Saharan Africa and implications for control: an analysis of data across geographical scales. *Lancet* 2018; published online March 1. http://dx.doi.org/10.1016/S0140-6736(17)33050-7.

Web Appendix to Mapping the Burden of Cholera in Africa and Implications for Control

Justin Lessler, Sean M. Moore, Francisco J. Luquero, Heather S. McKay, Rebecca Grais, Myriam Henkens, Martin Mengel, Jessica Dunoyer, Maurice M'bangombe, Elizabeth C. Lee, Mamoudou Harouna Djingarey, Bertrand Sudre, Didier Bompangue, Robert S.M. Fraser, Abdinasir Abubakar, William Perea, Dominique Legros, Andrew. S. Azman

correspondence to: justin@jhu.edu and azman@jhu.edu

Cholera data

Cholera data from 2010 to 2016 were obtained from multiple sources, including the World Health Organization (WHO), Médecins Sans Frontières, ProMED, situation reports from ReliefWeb and other websites, several Ministries of Health, and the scientific literature. Annual case counts reported to the WHO from 2010-2015 were included for each country in sub-Saharan Africa.[1] In addition, WHO provided additional sub-national reporting data for several countries. After detailed review with collaborators at the WHO, we attempted to contact key countries directly to obtain sub-national data and contextual information that might not be available from the WHO. We received sub-national reporting data directly from the Ministries of Health of Benin, Democratic Republic of Congo, Cameroon, Malawi, Mozambique, Nigeria and South Sudan. Médecins Sans Frontières and Epicentre provided cholera data from outbreaks, and publicly available reporting data were also obtained for refugee camps managed by the UN Refugee Agency (UNHCR).[2] Weekly cholera cases for West and Central Africa were obtained from United Nations Children's Fund (UNICEF).[3] Cholera reporting data for Madagascar were obtained from publicly available sources,[4] and publicly available cholera outbreak reports were obtained from ReliefWeb and ProMED.[5] Throughout the data collation process, the data entry team worked with data contributors ensure that locations and times with there were zero reported cases were reported as such, as opposed to missing data. Our analysis included a total of 279 datasets representing epidemiological time series spanning 1-7 years for a particular country (Table S1). All data was input into a standard schema that allows for flexible entry of data spanning multiple reporting periods and case definitions (see Table S2). These datasets include one or more observations for 2,283 different, though sometimes nested, locations in 37 countries (Figure S1). These locations included national-level observations for 35 different countries, 239 first-level administrative units (based on GADM subdivision classifications[6]), 1699 second-level administrative units, and 310 locations that were third-level administrative units or some other finer-scale location than a second-level administrative unit. Of these observations from the finest spatial-scale, 87% (270/310) were from locations contained within a single 20x20km grid-cell.

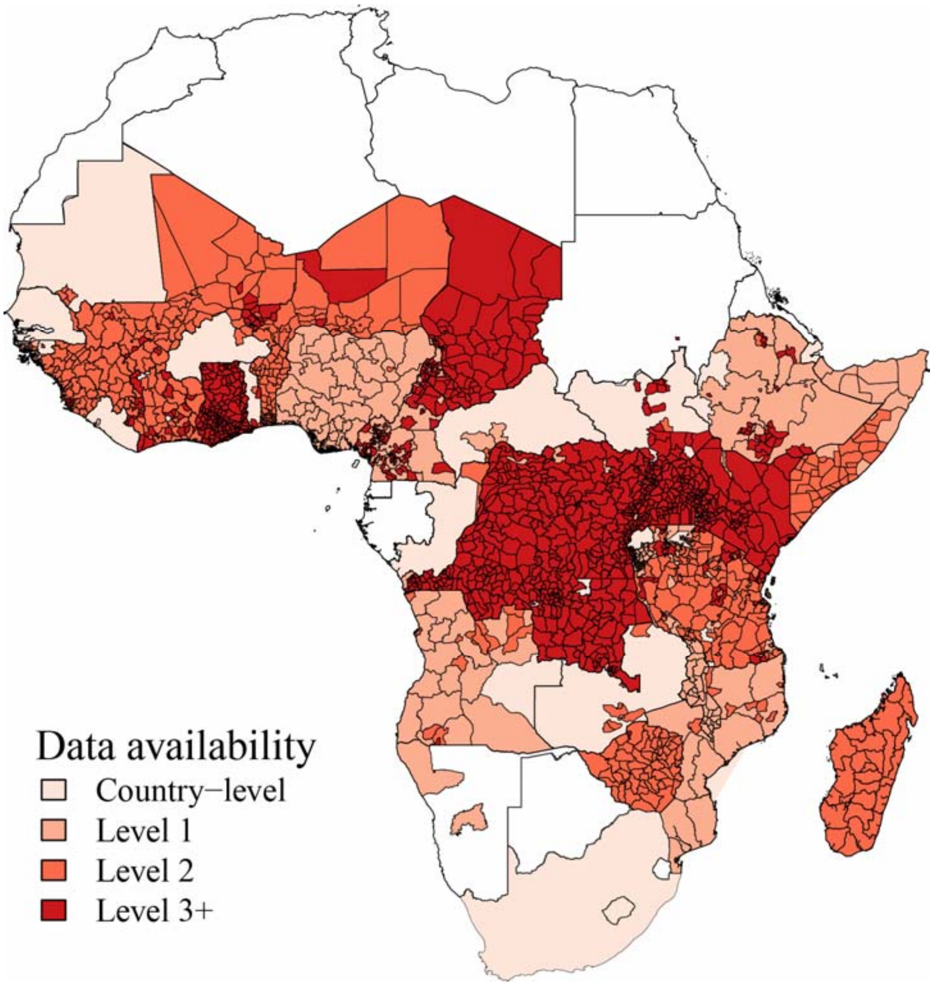

Fig. S1. Map of data on reported cholera cases from 2010-2016 included in generating the maps of cholera incidence. Color represents the lowest administrative level available for a given area.

Water and Sanitation Data

We included estimates of the median proportion of the population with access to improved water and sanitation as estimated by Pullan et al. (figures S2,S3).[7] The definition for access to improved drinking water was based on that of the WHO/UNICEF Joint Monitoring Program (JMP) [8] and included piped water into the dwelling; piped water to yard/plot/compound; public tap or standpipe; tubewell or borewell; protected dug well; protected spring; rainwater. Access to improved sanitation was based on a modified JMP definition and included flush toilets; piped sewer systems; septic tanks; ventilated improved pit latrines; pit latrines with slabs; composting toilets regardless of whether the facilities were shared. Pullan et al. did not include covariate information fro Djibouti, Eritrea and Botswana. Djibouti and Eritrea were excluded from the analysis, and covariate data for Botswana was obtained from WHO/UNICEF JMP 2014 progress report.[8]

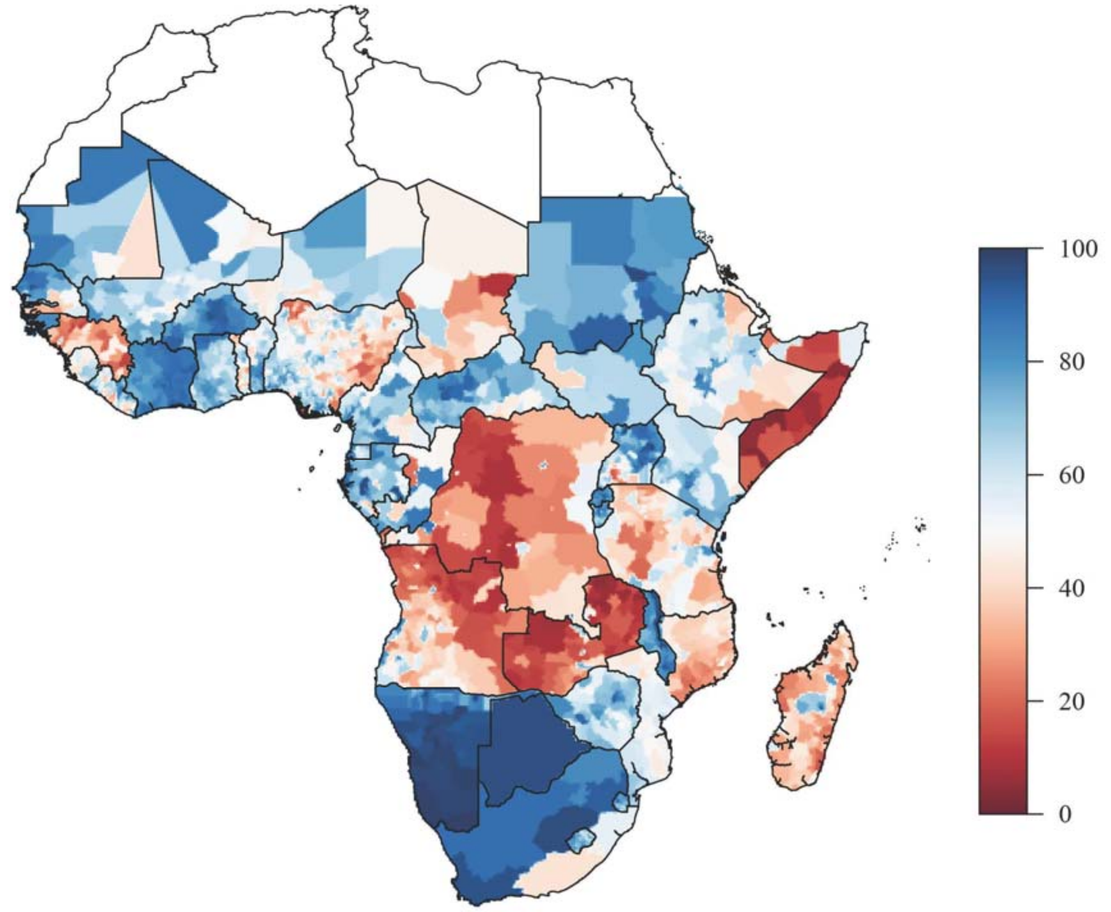

Fig. S2. Percentage of population with access to improved drinking water. Data from Pullan et al. 2014[1].

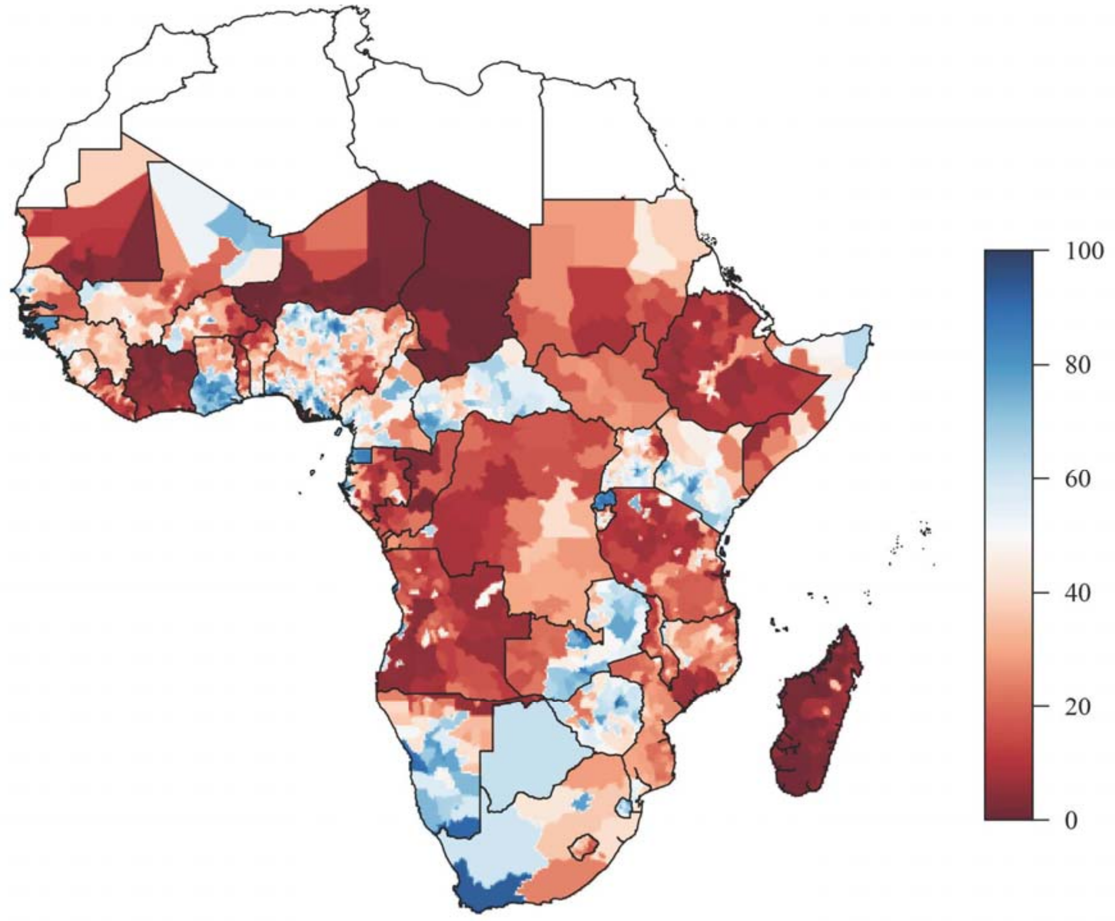

Fig S3. Percentage of population with access to improved sanitation. Data from Pullan et al. 2014[1].

Mapping Methodology

Data Processing and Assumptions

Multiple observations for the same geographic region covering different temporal periods or from different sources were treated as independent observations. Including annual observations over a seven-year period allowed us to estimate an average incidence without being too influenced by single outbreaks. Data from different, but overlapping, spatial scales were also treated as independent observations. We did not attempt to discriminate between data sources based on quality, so observations for the same region from different data sources were treated with equal weight. In several instances, multiple sources (e.g., national MoH and WHO) provided the same data. In these instances, the different sources were treated as independent observations so that cases would not be double-counted; however, because duplicate data are treated as independent observations these observations will have additional weighting in the model.

Modeling Framework

The entire study region (Sub-Saharan Africa) was divided into a total of 225,044 20 km by 20 km grid cells. We chose this grid cell size based on computational constraints and the spatial scale of the cholera and covariate data (e.g., information is lost in less than 11% of our cholera data by aggregating to this cell size). Grid cells falling outside of all observation areas and those with a population size of 0 (including grid cells covered by water) were excluded from analysis, resulting in N_j=61,795 included in the analysis. As a result of the process of spatial aggregation, grid cells that crossed borders of the study area (i.e., into water, countries not included, or zero population areas) only represent those portions of the grid cell lying with the study area. The annual cholera incidence in each grid cell, λ_j, was modeled using a log-linear regression,

$$\log(\lambda_j) = \beta_0 + \beta_p X_{p,j} + \psi_j,$$

with covariates, $X_{p,j}$, and spatially-correlated random effects, ψ_j. The random effects account for overdispersion and any unexplained spatial correlation in the data and were modeled by a conditional autoregressive (CAR) distribution.[9,10] Spatial correlation between random effects is determined by a binary N_j x N_j adjacency matrix, *A*, with element $a_{j,k}$ equal to one if grid cells (*j*,*k*) are neighbors (sharing an edge), and zero otherwise (and for *j*=*k*). The joint distribution of ψ is an N_j-dimensional multivariate normal distribution given by

$$\psi_j \sim N(0, \sigma_v^2 (D - \rho A)^{-1}),$$

where ρ is a parameter representing the relative strength of spatial dependence with $0 \leq \rho < 1$ and *D* is a diagonal matrix with entries $d_{j,j} = \sum_{k=1}^{N_j} a_{j,k}$, where $d_{j,j}$ represents the number of neighbors for grid cell *j* [11–13].

The expected number of cases, E_i, for each observation is the sum of the expected number of cases in each of N_i, grid cells included in the observation area, *i*:

$$E_i = \sum_{j=1}^{N_i} \lambda_j * p_j,$$

where p_j is the population size in grid cell j. Each observation, Y_i was mapped to the underlying grid cells that are within area i and were modeled by a Poisson process:

$$Y_i \sim \text{Pois}\left(E_i\right).$$

Grid cells were classified as within an observation area if the center of the grid cell fell within the observation polygon. If the grid cell was included within observation area *i*, then the entire population size for that grid cell was used in calculating the incidence and expected number of cases, E_i. Likewise, if the center of a grid cell fell outside of observation area *i*, then the grid cell was not included in the calculation for observation *i*, even if the grid cell was partially within area *i*.

The intercept term of the log-linear regression model, β_0 and the regression parameters $\beta = (\beta_1, \ldots, \beta_p)$ were assigned weakly informative Gaussian prior distributions, $\mathrm{N}\left(0, 10\right)$. The spatial autocorrelation parameter ρ was assigned a $\beta(2, 1)$ prior and the precision parameter τ_v from the spatial autocorrelation term ψ_j was assigned a $\Gamma(0.5, 0.0005)$ prior distribution. The covariates included in our analysis were proportion of population with access to improved drinking water, level of access to improved sanitation, population density, distance to the nearest coastline, and distance to the nearest major waterbody (covariate data sources described above and in the full text).

Categorization of Risk Levels

Based on a high-risk designation that was previously used in WHO recommendations and has since gained acceptance in the cholera community,[14] we designated areas (grid cells) with incidence greater than 1 in 1,000 as “high-risk” areas. We then designate the subsequent 10-fold decreases in risk (≥1 in 1,000 and <1 in 10,000; ≥1 in 10,000 and <1 in 100,000) as “moderate” and “mild” risk levels. Districts are designated to have the highest risk level experienced by 10% of their population or at least 100,000 residents (whichever is smaller).

Population over 1 year of Age

We assumed that 96.5% of the population was over 1 year of age based on an estimated birth rate of 37 per 1,000 people and an infant mortality rate of 56 per 1,000 births in sub-Saharan Africa for 2015.[15]

Additional Supplemental Figures and Tables:

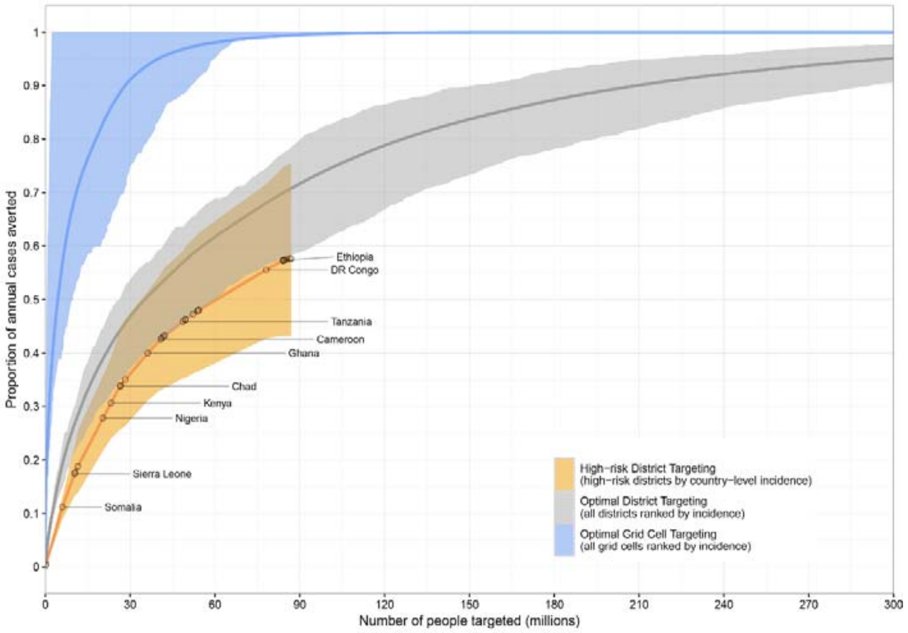

Fig. S4. Proportion of annual cholera cases in sub-Saharan Africa prevented as a function of the number of people targeted with a perfect intervention (or mix of interventions) under three different targeting strategies. The Optimal Grid Cell Targeting curve (blue) represents a strategy targeting all 20x20km grid cells in rank order by annual incidence. The Optimal District Targeting curve (green) represents a strategy targeting all districts in rank order by incidence regardless of country. The red curve represents a more realistic/practical strategy that targets all high-risk districts in each country at once, with countries ranked in order of mean incidence in high-risk districts. Bold lines represent the mean values and envelopes around each represent the 95% credible intervals. The results of the High-risk District Targeting strategy for all countries, including those not labeled in this figure, are ranked by mean annual incidence in Table S4.

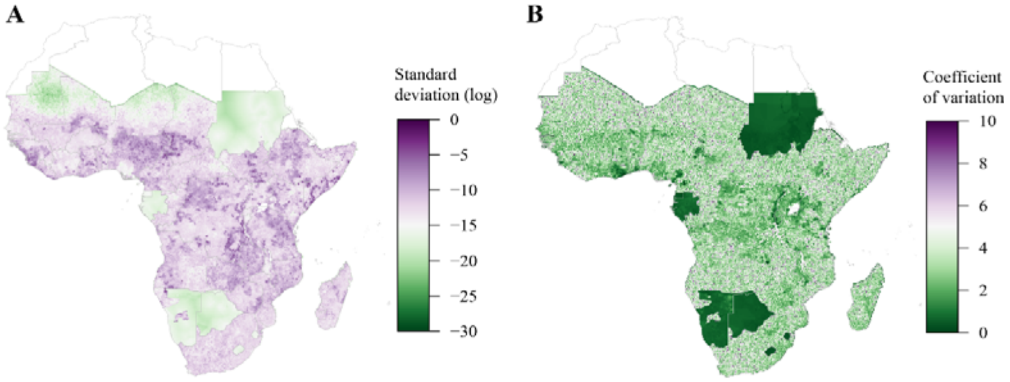

Fig. S5. (A) Standard deviation (log-scale), and (B) coefficient of variation of mean annual incidence from 2010-2016. Coefficient of variation is σ/μ.

Tables S1-S6

Table S1. Summary of cholera datasets by country. Number of locations is the number of unique locations (including national-level) per country, number of observations is number of reports per country, and number of years is the number of years from 2010 to 2016 with at least one report (including reporting 0).

Country	Number of locations	Number of observations	National level observation(s)	Admin 1 observations	Admin 2 observations	Admin 3+ observations	Number of years
Angola	41	115	Yes	17	22	1	6
Benin	81	414	Yes	3	77	0	7
Botswana	0	0	No	0	0	0	0
Burkina Faso	6	14	Yes	0	5	0	6
Burundi	44	96	Yes	13	27	3	7
Cameroon	145	189	Yes	11	0	133	7
Central African Rep	5	8	Yes	1	3	0	4
Chad	63	130	Yes	1	55	6	5
Cote d'Ivoire	80	239	Yes	0	50	29	7
Djibouti	1	3	Yes	0	0	0	3
DR Congo	522	2559	Yes	5	516	0	7
Eritrea	0	0	No	0	0	0	0
Ethiopia	39	56	Yes	8	1	29	3
Ghana	203	405	Yes	10	188	4	7
Guinea	54	161	Yes	1	47	5	7
Guinea-Bissau	31	39	Yes	7	23	0	5
Kenya	102	170	Yes	30	70	1	7
Liberia	3	10	Yes	1	1	0	7
Madagascar	23	47	Yes	0	22	0	3
Malawi	29	254	Yes	28	0	0	7
Mali	70	92	Yes	4	56	9	5
Mauritania	6	8	Yes	1	4	0	3
Mozambique	18	79	Yes	11	6	0	7
Namibia	2	5	No	2	0	0	2
Niger	58	200	Yes	7	35	15	7
Nigeria	39	210	Yes	37	1	0	7
Rep of Congo	4	14	Yes	1	2	0	5
Rwanda	2	2	Yes	0	1	0	2
Senegal	2	5	Yes	0	1	0	4
Sierra Leone	25	69	Yes	1	16	7	5
Somalia	54	172	Yes	10	43	0	7
South Africa	1	1	Yes	0	0	0	1
South Sudan	24	28	Yes	0	23	0	3
Sudan	1	1	No	0	0	1	1
Tanzania	202	711	Yes	28	149	24	6
Togo	7	17	Yes	0	4	2	7
Uganda	225	686	Yes	0	183	41	7
Zambia	9	12	Yes	0	8	0	4
Zimbabwe	62	98	Yes	1	60	0	6

Table S2: Simplified schema for the epidemiological data files used as basis for the included maps.

**Column Name**	**Description**	**Values**	**Required?**
Timing Block			
TL	Earliest possible time of "onset" as defined in this setting.	date in format: yyyy-mm-dd	yes
TR	Latest possible time of "onset" as defined in this setting/	date in format: yyyy-mm-dd	Yes
Location Block – included as appropriate for dataset			
who_region	WHO region for data	text	Yes
ISO_A1	ISO-3611-1 alpha 3 code for the country (see: http://en.wikipedia.org/wiki/ISO_3166-1_alpha-3)	text	Yes
ISO_A2_L1...ISO_A2_L*n*	ISO-3611-2 code or human readable name for the first through nth subdivision of the country (see: http://en.wikipedia.org/wiki/ISO_3166-2 and click on specific country code).	text	No
lat_case	Latitude of case household	text	No
long_case	Longitude of case household	text	No
Case Count Block – at least one column should be included, use all that are appropriate.			
Deaths	Number of individuals that died in this time frame	numeric	no
sCh	Number of suspected cholera cases during this time frame (Note: this includes all confirmed cases)	numeric	no
cCh	Number of confirmed cholera cases during this time frame	numeric	no
deaths_L	Left truncated deaths in this time frame (e.g. 3 would indicate less than 3 deaths)	numeric	no
deaths_R	Right truncated deaths in this time frame (e.g. 3 would indicate more than 3 deaths)	numeric	no
sCh_L	Left truncated suspected cases in this time frame (e.g. 3 would indicate less than 3 cases)	numeric	no
sCh_R	Right truncated suspected cases in this time frame (e.g. 3 would indicate more than 3 cases)	numeric	no
cCh_L	Left truncated confirmed cases in this time frame (e.g. 3 would indicate less than 3 cases)	numeric	no
cCh_R	Right truncated confirmed cases in this time frame (e.g. 3 would indicate more than 3 cases)	numeric	no

Table S3. Number of people living in districts with a subpopulation (>10% of total district population or >100,000 people) experiencing either high (≥1 per 1,000), moderate (1-10 per 10,000), or mild (1-10 per 100,000) cholera incidence. Based on 2010-2016 reported incidence estimates.

Country	>1 per 1,000 (95% CrI)		Percent Urban	>1-10 per 10,000 (95% CrI)		Percent Urban	>1-10 per 100,000 (95% CrI)		Percent Urban
Angola	395,696	(71,659 - 1,225,652)	0.0	2,172,756	(1,149,979 - 3,416,774)	7.1	6,785,005	(4,863,672 - 9,183,071)	28.9
Benin	64,297	(0 - 354,901)	0.0	2,171,992	(1,755,488 - 3,573,805)	32.7	3,648,779	(2,046,816 - 4,482,108)	42.4
Botswana	0	(0 - 0)	-	0	(0 - 0)	-	0	(0 - 0)	-
Burkina Faso	542,544	(0 - 542,544)	0.0	637,828	(0 - 1,814,332)	0.0	899,245	(261,417 - 2,026,682)	0.0
Burundi	645,212	(645,212 - 645,212)	26.8	1,160,209	(1,160,209 - 1,340,422)	0.0	3,803,091	(2,824,978 - 4,272,363)	28.1
Cameroon	4,598,018	(3,210,631 - 5,641,237)	18.1	11,121,613	(9,502,798 - 13,169,495)	52.8	4,714,523	(3,008,546 - 6,694,224)	5.8
Central African Rep	71,156	(0 - 92,801)	0.0	782,756	(0 - 1,125,356)	76.7	1,032,882	(555,702 - 2,057,084)	30.1
Chad	3,243,983	(2,114,280 - 4,489,554)	0.0	4,352,958	(3,084,465 - 5,667,122)	36.1	1,801,698	(523,693 - 3,138,723)	0.0
Cote d'Ivoire	1,049,005	(0 - 1,530,320)	16.2	531,067	(0 - 1,785,375)	56.9	6,989,860	(5,284,890 - 9,095,605)	83.7
DR Congo	23,856,539	(20,246,528 - 27,702,957)	16.3	20,302,237	(11,265,652 - 27,023,831)	14.9	20,780,506	(11,866,009 - 30,733,515)	44.4
Equatorial Guinea	0	(0 - 0)	-	0	(0 - 55,914)	-	19,815	(0 - 93,503)	0.0
Ethiopia	5,967,606	(2,537,817 - 11,625,989)	5.6	24,977,013	(10,540,410 - 43,998,996)	4.8	39,357,672	(26,343,649 - 56,561,168)	5.8
Gabon	0	(0 - 0)	-	0	(0 - 0)	-	0	(0 - 66,329)	-
Gambia	0	(0 - 0)	-	0	(0 - 0)	-	884,714	(884,714 - 1,047,872)	77.7
Ghana	7,976,232	(6,057,512 - 9,035,911)	73.2	7,558,433	(6,448,819 - 9,610,902)	49.5	9,815,452	(8,236,038 - 10,983,343)	52.4
Guinea	2,628,214	(2,395,007 - 3,294,971)	77.2	2,509,901	(1,456,481 - 3,222,728)	4.6	1,381,227	(628,119 - 2,385,200)	13.1
Guinea-Bissau	127,953	(66,099 - 197,252)	13.6	824,712	(683,116 - 976,700)	59.3	385,330	(143,394 - 706,245)	0.0
Kenya	2,843,874	(1,879,012 - 4,099,104)	6.7	13,527,956	(9,250,383 - 15,495,230)	46.3	11,841,405	(8,494,521 - 15,861,670)	24.2
Lesotho	0	(0 - 0)	-	0	(0 - 0)	-	0	(0 - 475,132)	-
Liberia	181,714	(99,856 - 434,411)	25.6	356,062	(34,271 - 688,772)	4.9	944,124	(304,576 - 2,630,765)	12.5
Madagascar	0	(0 - 0)	-	1,447,503	(0 - 5,693,778)	0.0	6,248,182	(1,151,538 - 9,401,647)	0.0
Malawi	464,185	(100,828 - 859,960)	0.1	2,649,135	(1,642,694 - 3,675,005)	2.7	4,617,165	(3,189,206 - 6,020,891)	19.1
Mali	146,167	(0 - 701,814)	0.0	2,182,132	(1,396,808 - 2,890,926)	11.4	2,032,784	(997,851 - 3,562,150)	2.3
Mauritania	47,214	(0 - 47,214)	0.0	0	(0 - 343,660)	-	561,781	(0 - 1,238,483)	0.4
Mozambique	1,626,425	(676,216 - 2,463,809)	20.0	3,043,176	(1,298,204 - 5,140,155)	23.8	11,411,133	(7,629,142 - 14,465,353)	16.5
Namibia	0	(0 - 342,420)	-	585,923	(30,709 - 697,403)	95.9	53,275	(0 - 190,410)	0.0
Niger	784,421	(0 - 2,064,362)	0.0	4,116,844	(1,877,464 - 6,557,361)	20.2	8,828,564	(5,713,605 - 12,101,652)	31.3
Nigeria	8,885,306	(6,690,121 - 14,121,092)	26.4	25,579,758	(16,481,900 - 33,318,331)	28.5	52,188,851	(40,570,405 - 68,386,349)	35.8
Rep of Congo	162,024	(0 - 241,054)	0.0	1,091,233	(941,834 - 1,354,593)	71.8	3,066,695	(2,821,721 - 3,440,891)	84.1
Rwanda	0	(0 - 0)	-	3,033,448	(3,033,448 - 3,033,448)	64.2	0	(0 - 1,269,600)	-
Senegal	0	(0 - 0)	-	0	(0 - 0)	-	337,052	(0 - 1,049,529)	0.0
Sierra Leone	4,306,623	(2,114,589 - 5,328,026)	38.7	1,809,803	(0 - 3,410,214)	0.0	379,344	(0 - 1,645,801)	0.0
Somalia	5,802,869	(4,643,745 - 6,624,358)	29.8	1,572,582	(548,393 - 2,473,992)	33.2	1,209,707	(412,485 - 2,220,609)	0.0
South Africa	0	(0 - 0)	-	0	(0 - 0)	-	1,846,563	(0 - 6,479,288)	23.5
South Sudan	979,262	(430,238 - 1,718,093)	11.8	2,026,680	(859,723 - 3,190,980)	0.0	3,711,172	(1,867,953 - 5,779,698)	1.4
Sudan	0	(0 - 0)	-	0	(0 - 0)	-	0	(0 - 288,193)	-
Swaziland	0	(0 - 0)	-	0	(0 - 39,164)	-	25,835	(0 - 128,002)	0.0
Tanzania	6,803,857	(4,938,927 - 9,175,220)	29.6	21,472,692	(17,788,793 - 24,803,103)	19.0	13,087,493	(10,281,035 - 16,255,522)	8.8

Togo	621,488	(0 - 621,488)	23.6	1,021,469	(789,225 - 2,531,263)	79.2	3,938,539	(1,357,867 - 5,651,561)	32.2
Uganda	1,492,246	(1,389,935 - 1,941,594)	0.0	5,613,923	(4,686,142 - 6,840,392)	0.3	10,153,474	(8,309,450 - 12,743,846)	38.9
Zambia	426,062	(0 - 997,509)	5.5	5,586,221	(4,301,358 - 7,018,562)	48.3	8,445,417	(6,266,698 - 10,387,379)	19.4
Zimbabwe	443,201	(0 - 716,815)	0.0	1,738,821	(785,848 - 2,685,814)	0.0	5,152,788	(2,189,091 - 6,668,098)	38.3
**Total**	**87,183,393**	**(60,308,212 - 118,877,644)**		**177,558,836**	**(112,794,614 - 248,663,898)**		**252,381,142**	**(169,028,781 - 351,869,554)**	

Table S4. Districts (ISO administrative level 2) with a subpopulation (>10% of total district population or >100,000 people) where mean annual incidence ≥ 1 per 1,000. Frequency is percent of iterations where the district meets this high-risk threshold. All districts with a frequency of ≥ 50% are listed.

Country	ISO level 1	ISO level 2	Population	Mean Incidence per 1,000 (95% CrI)	Percent of Iterations
Benin	Atakora	Toucountouna	64,297	0.45 (0.26 - 0.59)	74.6
Burkina Faso	Centre-Est	Koulpélogo	542,544	0.17 (0.00 - 0.21)	70.6
Burundi	Bubanza	Gihanga	136,418	0.85 (0.80 - 0.89)	100
Burundi	Bujumbura Mairie	Buterere	47,796	0.68 (0.63 - 0.73)	100
Burundi	Bujumbura Rural	Mutimbuzi	189,321	0.70 (0.66 - 0.73)	100
Burundi	Bururi	Rumonge	167,339	0.73 (0.59 - 0.88)	100
Burundi	Makamba	Nyanza-Lac	104,338	0.58 (0.52 - 0.64)	99.9
Cameroon	Extrême-Nord	Mayo Danay	862,154	1.53 (1.12 - 1.76)	100
Cameroon	Extrême-Nord	Mayo Tsanaga	1,145,127	0.63 (0.59 - 0.88)	100
Cameroon	Littoral	Moungo	798,548	0.64 (0.07 - 0.68)	81.9
Cameroon	Nord	Mayo Louti	588,839	0.62 (0.43 - 1.09)	53.1
Cameroon	Ouest	Noun	631,208	0.50 (0.47 - 0.52)	84.1
Cameroon	Sud-Ouest	Fako	572,141	1.41 (1.37 - 1.45)	100
Central African Rep	Lobaye	Mongoumba	71,156	0.84 (0.70 - 1.22)	59.5
Chad	Lac	Wayi	226,175	0.33 (0.23 - 0.96)	77.9
Chad	Mayo-Kebbi Est	Kabbia	277,914	1.54 (0.70 - 2.76)	99.8
Chad	Mayo-Kebbi Est	Mayo-Boneye	663,182	2.75 (1.24 - 2.95)	100
Chad	Mayo-Kebbi Ouest	Lac Léré	452,013	1.81 (1.55 - 2.50)	100
Chad	Mayo-Kebbi Ouest	Mayo-Dallah	459,669	0.94 (0.49 - 1.53)	74.9
Chad	Ouaddaï	Djourf Al Ahmar	131,114	0.56 (0.00 - 0.74)	50.5
Chad	Salamat	Barh Azoum	231,205	1.11 (1.00 - 1.20)	100
Cote d'Ivoire	Comoé	Sud Comoé	1,049,005	0.14 (0.10 - 0.16)	73.3
DR Congo	Bandundu	Mai-Ndombe	1,686,780	0.50 (0.48 - 0.52)	61.4
DR Congo	Katanga	Haut-Lomami	3,103,217	0.54 (0.52 - 0.55)	100
DR Congo	Katanga	Tanganika	2,159,637	0.81 (0.77 - 0.89)	61
DR Congo	Kivu	Nord-Kivu	6,492,238	0.44 (0.43 - 0.45)	100
DR Congo	Kivu	Sud-Kivu	5,148,193	0.79 (0.76 - 0.80)	100
DR Congo	Orientale	Ituri	5,502,881	0.18 (0.17 - 0.19)	100
Ghana	Ashanti	Ejura Sekyedumase	94,672	0.57 (0.40 - 0.72)	88.9
Ghana	Brong Ahafo	Atebubu-Amantin	79,602	2.47 (2.21 - 2.62)	100
Ghana	Brong Ahafo	Pru	113,105	0.87 (0.74 - 1.02)	100
Ghana	Central	Awutu Efutu Senya	385,613	1.23 (1.20 - 1.26)	100
Ghana	Central	Komenda-Edina-Eguafo-Abirem	159,941	4.48 (4.12 - 4.83)	100
Ghana	Central	Lower Denkyira	136,749	0.23 (0.20 - 0.27)	99.6
Ghana	Eastern	Akwapim North	201,511	0.50 (0.47 - 0.52)	100
Ghana	Eastern	Akwapim South	219,823	0.93 (0.90 - 0.96)	100
Ghana	Eastern	Birim North	198,538	0.31 (0.28 - 0.34)	62.2
Ghana	Greater Accra	Accra	2,104,384	1.15 (1.13 - 1.16)	100
Ghana	Greater Accra	Dangbe East	255,709	6.31 (6.18 - 6.45)	100
Ghana	Greater Accra	Ga West	699,023	1.53 (1.51 - 1.55)	100
Ghana	Northern	Bole	71,255	1.05 (0.80 - 1.24)	54.5
Ghana	Upper East	Bawku Municipal	362,310	0.18 (0.15 - 0.29)	70.6
Ghana	Upper East	Garu Tempane	220,579	0.20 (0.02 - 0.24)	70.6
Ghana	Volta	Hohoe	287,130	0.48 (0.41 - 0.55)	99.9
Ghana	Volta	Ketu	1,947,068	0.07 (0.07 - 0.08)	94.6
Ghana	Volta	Nkwanta	283,412	0.40 (0.35 - 0.46)	84.6
Ghana	Western	Jomoro	185,936	0.38 (0.32 - 0.44)	82.6
Guinea	Conakry	Conakry	1,622,111	0.50 (0.49 - 0.51)	100
Guinea	Kindia	Coyah	491,222	0.75 (0.63 - 0.80)	100
Guinea	Kindia	Dubréka	281,674	0.66 (0.53 - 0.98)	100
Guinea-Bissau	Biombo	Quinhamel	59,011	3.46 (3.23 - 3.71)	100
Guinea-Bissau	Bolama	Bolama	7,088	24.36 (0.00 - 28.68)	84.3
Kenya	Elgeyo-Marakwet	Marakwet East	101,472	0.26 (0.00 - 0.67)	52.9
Kenya	Elgeyo-Marakwet	Marakwet West	87,460	0.23 (0.02 - 0.36)	70.6

Kenya	Embu	Mbeere South	183,005	0.41	(0.19	- 0.58)	65.2
Kenya	Garissa	Daadab	181,917	3.73	(1.56	- 3.94)	100
Kenya	Garissa	Garissa Township	37,186	2.00	(0.00	- 2.47)	50.5
Kenya	Homa Bay	Mbita	155,229	1.17	(0.00	- 1.56)	50.5
Kenya	Mandera	Mandera East	163,660	3.56	(1.20	- 7.19)	100
Kenya	Marsabit	Moyale	183,708	0.82	(0.74	- 0.92)	75.3
Kenya	Marsabit	Saku	52,694	0.46	(0.00	- 1.44)	71.8
Kenya	Migori	Kuria East	116,829	1.13	(0.97	- 4.01)	100
Kenya	Migori	Nyatike	303,474	1.58	(0.01	- 1.94)	90.4
Kenya	Tana River	Bura	190,019	1.47	(0.00	- 1.82)	50.5
Kenya	Tana River	Garsen	98,603	0.74	(0.00	- 4.46)	53.2
Kenya	Tharaka-Nithi	Tharaka	106,418	1.81	(0.84	- 2.93)	78.9
Kenya	Wajir	Wajir South	167,631	3.50	(2.88	- 3.66)	100
Kenya	West Pokot	Pokot South	156,371	0.59	(0.06	- 1.26)	71.9
Kenya	West Pokot	Sigor	91,006	1.20	(0.00	- 3.09)	73.1
Malawi	Nsanje	TA Mlolo	76,328	0.69	(0.00	- 1.17)	57.8
Mauritania	Gorgol	Monguel	47,214	0.45	(0.32	- 0.59)	92.3
Mozambique	Cabo Delgado	Montepuez	266,107	1.99	(0.00	- 2.25)	76
Mozambique	Nassa	Lichinga	410,109	0.93	(0.01	- 0.98)	80.1
Mozambique	Zambezia	Nicoadala	532,312	1.00	(0.03	- 1.38)	53.1
Niger	Tillabéry	Tillabéry	353,569	1.22	(1.01	- 1.27)	68.6
Nigeria	Adamawa	Michika	240,248	2.01	(1.37	- 2.58)	100
Nigeria	Gombe	Gombe	4,849	1.18	(0.02	- 1.40)	72.5
Nigeria	Gombe	Kwami	285,017	0.33	(0.26	- 0.56)	89.1
Nigeria	Gombe	Yamaltu	332,867	0.32	(0.12	- 1.64)	69.3
Nigeria	Kaduna	Chikun	623,295	0.20	(0.06	- 1.11)	58.7
Nigeria	Katsina	Dandume	147,941	0.51	(0.00	- 1.98)	52.2
Nigeria	Yobe	Borsari	256,181	0.87	(0.00	- 1.26)	52.1
Nigeria	Yobe	Karasuwa	107,569	0.36	(0.00	- 0.52)	52.1
Nigeria	Yobe	Yusufari	208,908	0.35	(0.00	- 0.55)	52.1
Rep of Congo	Plateaux	Gamboma	162,024	1.82	(0.00	- 2.73)	60
Sierra Leone	Eastern	Kenema	655,808	0.61	(0.46	- 0.71)	51.4
Sierra Leone	Northern	Bombali	547,082	1.04	(0.95	- 1.23)	100
Sierra Leone	Northern	Kambia	426,200	1.81	(1.43	- 1.91)	58.7
Sierra Leone	Northern	Port Loko	1,061,309	1.72	(1.49	- 5.58)	63.2
Sierra Leone	Northern	Tonkolili	417,512	0.98	(0.88	- 1.24)	66.8
Sierra Leone	Southern	Bo	610,650	0.47	(0.40	- 0.66)	59.6
Sierra Leone	Southern	Bonthe	177,691	0.54	(0.44	- 1.31)	60.3
Sierra Leone	Southern	Moyamba	351,607	0.86	(0.55	- 1.70)	99.1
Sierra Leone	Southern	Pujehun	290,468	1.50	(1.12	- 1.68)	71.4
Sierra Leone	Western	Western Rural	401,195	9.13	(8.80	- 9.30)	100
Sierra Leone	Western	Western Urban	388,505	3.91	(3.78	- 5.62)	100
Somalia	Bakool	Tiyeeglow	105,067	3.74	(3.55	- 3.89)	100
Somalia	Banaadir	Mogadisho	932,006	4.53	(4.48	- 4.57)	100
Somalia	Bari	Bosaaso	286,768	4.04	(0.00	- 4.32)	74.5
Somalia	Bay	Baydhabo	477,504	1.46	(1.42	- 1.52)	95.7
Somalia	Bay	Buur Xakaba	175,626	0.93	(0.84	- 1.02)	61.7
Somalia	Bay	Diinsoor	109,035	1.44	(1.12	- 1.62)	53.5
Somalia	Galguduud	Caabudwaaq	129,366	0.78	(0.00	- 0.84)	76
Somalia	Galguduud	Cadaado	61,825	3.10	(2.89	- 4.89)	76
Somalia	Gedo	Baar-Dheere	154,471	0.70	(0.64	- 0.76)	99.9
Somalia	Gedo	Beled Xaawo	138,724	3.30	(1.90	- 7.49)	100
Somalia	Gedo	Dolow	83,177	1.46	(1.30	- 2.14)	100
Somalia	Gedo	Garbahaaray	82,395	1.98	(1.78	- 3.43)	54.2
Somalia	Gedo	Luuk	95,695	2.12	(1.95	- 2.28)	100
Somalia	Hiiraan	Buulo Burdo	174,397	1.24	(1.12	- 2.87)	51.2
Somalia	Hiiraan	Jalalaqsi	74,007	8.91	(5.46	- 9.53)	73.6
Somalia	Jubbada Dhexe	Bu'aale	73,956	6.78	(3.92	- 8.89)	58.8
Somalia	Jubbada Dhexe	Jilib	144,479	4.16	(3.04	- 5.74)	95.6
Somalia	Jubbada Dhexe	Saakow	98,750	7.00	(5.53	- 7.46)	100
Somalia	Jubbada Hoose	Afmadow	140,533	2.28	(1.44	- 3.08)	100
Somalia	Jubbada Hoose	Badhaadhe	70,167	4.53	(2.88	- 5.10)	63.1
Somalia	Jubbada Hoose	Jamaame	170,639	3.60	(3.09	- 5.26)	99.3

Somalia	Jubbada Hoose	Kismaayo	212,102	4.65	(4.05	- 5.25)	100
Somalia	Mudug	Gaalkacayo	226,200	0.86	(0.79	- 0.94)	78.9
Somalia	Mudug	Xarardheere	85,863	8.62	(6.00	- 9.38)	64.7
Somalia	Nugaal	Garoowe	84,006	7.84	(0.00	- 9.04)	51.3
Somalia	Shabeellaha Dhexe	Balcad	306,715	1.58	(1.27	- 2.08)	100
Somalia	Shabeellaha Dhexe	Jawhar	288,094	1.16	(0.65	- 1.32)	77
Somalia	Shabeellaha Hoose	Afgooye	707,610	1.44	(1.41	- 1.48)	100
Somalia	Shabeellaha Hoose	Baraawe	100,427	1.42	(1.32	- 1.58)	90.6
Somalia	Shabeellaha Hoose	Kuntuwaaray	78,175	0.98	(0.79	- 1.46)	100
Somalia	Shabeellaha Hoose	Marka	257,489	1.70	(1.49	- 1.81)	100
South Sudan	Central Equatoria	Bahr al Jabal	549,024	2.66	(2.15	- 2.74)	53.2
South Sudan	Unity	Rabkona	351,029	1.72	(1.60	- 1.82)	85.8
Tanzania	Dar es Salaam	Ilala	740,148	0.63	(0.44	- 0.67)	100
Tanzania	Dar es Salaam	Temeke	948,228	0.45	(0.23	- 0.54)	61.3
Tanzania	Mara	Butiama	364,721	0.33	(0.28	- 0.85)	71.2
Tanzania	Mara	Musoma Rural	517,852	1.71	(0.78	- 1.89)	92.4
Tanzania	Mara	Rorya	569,757	0.21	(0.18	- 0.24)	66.4
Tanzania	Mara	Tarime	526,917	0.33	(0.29	- 1.07)	100
Tanzania	Morogoro	Gairo	111,293	2.34	(1.66	- 3.06)	82.9
Tanzania	Mwanza	Ukerewe	548,720	0.25	(0.23	- 0.35)	88.2
Tanzania	Pwani	Kisarawe	279,281	0.95	(0.90	- 1.14)	100
Tanzania	Simiyu	Busega	266,550	0.55	(0.45	- 0.69)	91.9
Tanzania	Singida	Mkalama	227,853	0.31	(0.23	- 0.48)	66
Tanzania	Singida	Singida Rural	163,225	0.89	(0.58	- 1.21)	97.7
Tanzania	Tanga	Handeni	298,894	0.82	(0.62	- 0.92)	98.7
Tanzania	Tanga	Handeni Township Authority	39,932	1.89	(1.45	- 3.06)	100
Tanzania	Tanga	Korogwe	363,818	0.36	(0.30	- 0.45)	99.3
Tanzania	Tanga	Korogwe Township Authority	45,456	0.94	(0.84	- 1.11)	99.3
Togo	Plateaux	Kloto	621,488	0.22	(0.01	- 0.29)	60.4
Uganda	Kalangala	Bujumba	9,192	0.53	(0.00	- 1.14)	56.3
Uganda	Kisoro	Kisoro	707,074	0.29	(0.28	- 0.31)	100
Uganda	Kyenjojo	Kyaka	192,297	0.35	(0.32	- 0.37)	98.9
Uganda	Nebbi	Padyere	336,750	0.70	(0.67	- 0.74)	100
Zimbabwe	Manicaland	Chipinge	443,201	0.38	(0.34	- 0.43)	79.4

Table S5. Number of people living in high-risk districts (as defined in the text) for each country, ranked by the mean annual incidence in these high-risk districts. Ranking by cases represents the order of countries by mean number of cases as presented in Fig 3 of the main text.

Country	Population in High-risk Districts		Mean Incidence (per 1,000)		Mean Annual Cases		Ranking by Cases
Guinea-Bissau	127,953	(66,099 - 197,252)	3.3	(2.5 - 5.8)	424	(381 - 485)	17
Somalia	5,802,869	(4,643,745 - 6,624,358)	2.9	(2.9 - 3.0)	16,663	(13,735 - 18,967)	1
Sierra Leone	4,306,623	(2,114,589 - 5,328,026)	2.2	(2.8 - 3.1)	9,641	(6,644 - 14,882)	4
Liberia	181,714	(99,856 - 434,411)	1.9	(0.9 - 2.9)	352	(288 - 389)	18
South Sudan	979,262	(430,238 - 1,718,093)	1.8	(1.6 - 2.4)	1,748	(701 - 4,063)	11
Nigeria	8,885,306	(6,690,121 - 14,121,092)	1.6	(1.1 - 1.8)	13,958	(12,113 - 15,929)	2
Kenya	2,843,874	(1,879,012 - 4,099,104)	1.5	(1.3 - 1.9)	4,378	(3,549 - 5,212)	7
Chad	3,243,983	(2,114,280 - 4,489,554)	1.5	(1.3 - 1.9)	4,959	(2,712 - 8,374)	6
Rep of Congo	162,024	(0 - 241,054)	1.5	(0.0 - 2.3)	246	(0 - 559)	22
Mozambique	1,626,425	(676,216 - 2,463,809)	1.0	(0.9 - 1.4)	1,668	(962 - 2,252)	12
Ghana	7,976,232	(6,057,512 - 9,035,911)	1.0	(0.9 - 1.2)	7,678	(7,388 - 7,989)	5
Cameroon	4,598,018	(3,210,631 - 5,641,237)	0.9	(0.9 - 1.0)	4,046	(3,174 - 4,931)	9
Angola	395,696	(71,659 - 1,225,652)	0.9	(0.5 - 0.6)	341	(39 - 767)	19
Burundi	645,212	(645,212 - 645,212)	0.8	(0.7 - 0.8)	440	(416 - 462)	16
Zambia	426,062	(0 - 997,509)	0.6	(0.0 - 0.7)	268	(0 - 739)	20
Tanzania	6,806,437	(4,941,507 - 9,177,800)	0.6	(0.5 - 0.6)	4,078	(2,949 - 4,831)	8
Niger	784,421	(0 - 2,064,362)	0.6	(0.0 - 0.6)	484	(0 - 1,152)	15
Benin	64,297	(0 - 354,901)	0.6	(0.0 - 0.3)	40	(0 - 99)	28
Guinea	2,628,214	(2,395,007 - 3,294,971)	0.6	(0.6 - 0.7)	1,599	(1,335 - 2,230)	13
Uganda	1,492,246	(1,389,935 - 1,941,594)	0.6	(0.4 - 0.6)	852	(563 - 1,099)	14
Malawi	464,185	(100,828 - 859,960)	0.5	(0.7 - 0.8)	254	(85 - 642)	21
Central African	71,156	(0 - 92,801)	0.5	(0.0 - 0.9)	39	(0 - 88)	29
DR Congo	23,856,539	(20,246,528 - 27,702,957)	0.5	(0.5 - 0.5)	11,668	(9,492 - 14,138)	3
Ethiopia	5,967,606	(2,537,817 - 11,625,989)	0.5	(0.3 - 0.5)	2,897	(1187 - 5,432)	10
Mali	146,167	(0 - 701,814)	0.4	(0.0 - 0.3)	65	(0 - 205)	27
Mauritania	47,214	(0 - 47,214)	0.4	(0.0 - 0.6)	20	(0 - 28)	30
Zimbabwe	443,201	(0 - 716,815)	0.3	(0.0 - 0.3)	145	(0 - 241)	24
Togo	621,488	(0 - 621,488)	0.2	(0.0 - 0.3)	94	(0 - 183)	25
Cote d'Ivoire	1,049,005	(0 - 1,530,320)	0.1	(0.0 - 0.2)	150	(0 - 299)	23
Burkina Faso	542,544	(0 - 542,544)	0.1	(0.0 - 0.2)	70	(0 - 114)	26

Table S6. County cholera dynamics as characterized by annual cholera incidence and the coefficient of variation in incidence (CoV) as reported to WHO for 2000-2015. Countries are ordered from lowest CoV (i.e., most "endemic") to highest CoV (i.e., most "epidemic").

Country	Years Reporting	Mean Annual Cases	Mean Annual Incidence (per 100K)	CoV
DR Congo	16	21,268	25.2	0.39
Sudan	4	18,829	45.9	0.43
Burundi	16	700	4.8	0.58
Djibouti	4	1,272	136.1	0.68
Uganda	15	2,632	6.3	0.75
South Sudan	2	4,120	31.4	0.79
Mozambique	16	9,064	29.1	0.86
Tanzania	15	5,059	9.0	0.95
Zambia	13	3,415	19.1	0.99
Mali	11	915	4.9	1.14
Benin	14	813	6.6	1.16
Niger	15	1,150	5.2	1.21
Botswana	4	6	0.3	1.26
Ethiopia	7	16,466	16.3	1.27
Togo	16	523	5.9	1.31
Rwanda	10	404	2.7	1.32
Nigeria	16	9,953	5.4	1.32
Burkina Faso	8	289	1.5	1.35
Chad	9	4,035	26.1	1.39
Central African Rep	5	92	1.7	1.49
Kenya	13	2,870	5.7	1.51
Guinea	14	1,881	13.9	1.52
Somalia	14	13,744	120.4	1.56
Ghana	16	4,535	14.8	1.60
Equatorial Guinea	3	2,150	245.6	1.71
Gambia	4	57	2.4	1.85
Angola	10	10,959	41.9	1.88
Côte d'Ivoire	15	928	3.9	1.88
Rep of Congo	9	1,309	16.7	1.91
Cameroon	16	3,144	12.1	1.92
Madagascar	6	6,093	25.1	1.93
Eritrea	4	30	0.5	1.98
Guinea-Bissau	11	4,088	184.0	1.99
South Africa	10	16,036	28.9	2.01
Sierra Leone	7	4,123	58.0	2.05
Namibia	7	620	22.1	2.06
Malawi	14	3,949	20.9	2.13
Gabon	5	127	6.8	2.20
Liberia	15	3,816	66.2	2.27
Mauritania	6	720	13.8	2.32
Zimbabwe	14	9,886	61.3	2.33
Senegal	9	4,288	25.6	2.42
Swaziland	10	703	45.9	2.50

References

1 WHO | Weekly epidemiological record: cholera articles. 2014; published online Aug 20. http://www.who.int/cholera/statistics/en/ (accessed March 30, 2016).

2 UNHCR. Twine. http://twine.unhcr.org (accessed March 30, 2016).

3 WCAR Epidemiological Updates | Cholera | UNICEF. UNICEF. http://www.unicef.org/cholera/index_74805.html (accessed April 6, 2016).

4 Instat M. Annuaire statistique du secteur sante de Madagascar. http://instat.mg/sante/annuaire-statistique-du-secteur-sante-de-madagascar-1999-a-2013 (accessed March 30, 2016).

5 ReliefWeb. http://reliefweb.int (accessed March 30, 2016).

6 Hijmans R, Garcia N, Wieczorek J. GADM: database of global administrative areas. 2010.

7 Pullan RL, Freeman MC, Gething PW, Brooker SJ. Geographical inequalities in use of improved drinking water supply and sanitation across Sub-Saharan Africa: mapping and spatial analysis of cross-sectional survey data. *PLoS Med* 2014; **11**: e1001626.

8 UNICEF - Progress on drinking water and sanitation W, 2012. WHO Joint Monitoring Programme for Water Supply and Sanitation. 2012.

9 Besag J, Julian B, Jeremy Y, Annie M. Bayesian image restoration, with two applications in spatial statistics. *Ann Inst Stat Math* 1991; **43**: 1–20.

10 Banerjee S, Carlin BP, Gelfand AE. Hierarchical Modeling and Analysis for Spatial Data, Second Edition. CRC Press, 2014.

11 Stern HS, Cressie N. Posterior predictive model checks for disease mapping models. *Stat Med* 2000; **19**: 2377–97.

12 White G, Gentry W, Ghosh SK. A stochastic neighborhood conditional autoregressive model for spatial data. *Comput Stat Data Anal* 2009; **53**: 3033–46.

13 Lee D. A comparison of conditional autoregressive models used in Bayesian disease mapping. *Spat Spatiotemporal Epidemiol* 2011; **2**: 79–89.

14 Meeting of the Strategic Advisory Group of Experts on immunization, October 2009 - conclusions and recommendations. *Wkly Epidemiol Rec* 2009; **84**: 517–32.

15 Bank W. World Development Indicators 2015. World Bank Publications, 2015.
